# Bone Stress Injuries in Runners Using Carbon Fiber Plate Footwear

**DOI:** 10.1007/s40279-023-01818-z

**Published:** 2023-02-13

**Authors:** Adam Tenforde, Tim Hoenig, Amol Saxena, Karsten Hollander

**Affiliations:** 1 Spaulding Rehabilitation Hospital, Department of Physical Medicine and Rehabilitation, Harvard Medical School, Charlestown, MA USA; 2 Department of Trauma and Orthopaedic Surgery, University Medical Center Hamburg-Eppendorf, Hamburg, Germany; 3 Department of Sports Medicine, Sutter-PAMF, Palo Alto, CA USA; 4 Institute of Interdisciplinary Exercise Science and Sports Medicine, MSH Medical School Hamburg, Am Kaiserkai 1, 20457 Hamburg, Germany

## Abstract

The introduction of carbon fiber plate footwear has led to performance benefits in runners. The mechanism for these changes in running economy includes altered biomechanics of the foot and ankle. The association of this footwear with injuries has been a topic of debate clinically, but not described in the literature. In this Current Opinion article, illustrated by a case series of five navicular bone stress injuries in highly competitive running athletes, we discuss the development of running-related injuries in association with the use of carbon fiber plate footwear. While the performance benefits of this footwear are considerable, sports medicine providers should consider injuries possibly related to altered biomechanical demands affecting athletes who use carbon fiber plate footwear. Given the introduction of carbon fiber plate footwear into athletics and other endurance sports, strategies may be required to reduce risk of injury due to altered foot and ankle mechanics. This article is intended (1) to raise awareness on possible health concerns around the use of carbon fiber plate footwear, (2) to suggest a slow gradual transition from habitual to carbon fiber plate footwear, and (3) to foster medical research related to carbon fiber plate technology and injuries.

## Key Points

**Table:** 

The benefits of carbon fiber plate footwear have been documented in the scientific literature and are well accepted in the track and field and road racing community.
Prior reports of injuries using this technology have been observed clinically; these concerns have not been documented in the literature and limit knowledge among medical providers concerning possible association with development of injuries.
This Current Opinion article including a case series of navicular bone stress injuries after using carbon fiber plate footwear is intended to raise awareness that health concerns around use of carbon fiber plate footwear should be considered when athletes adopt this new footwear.

## Background

**Fig. 1:**
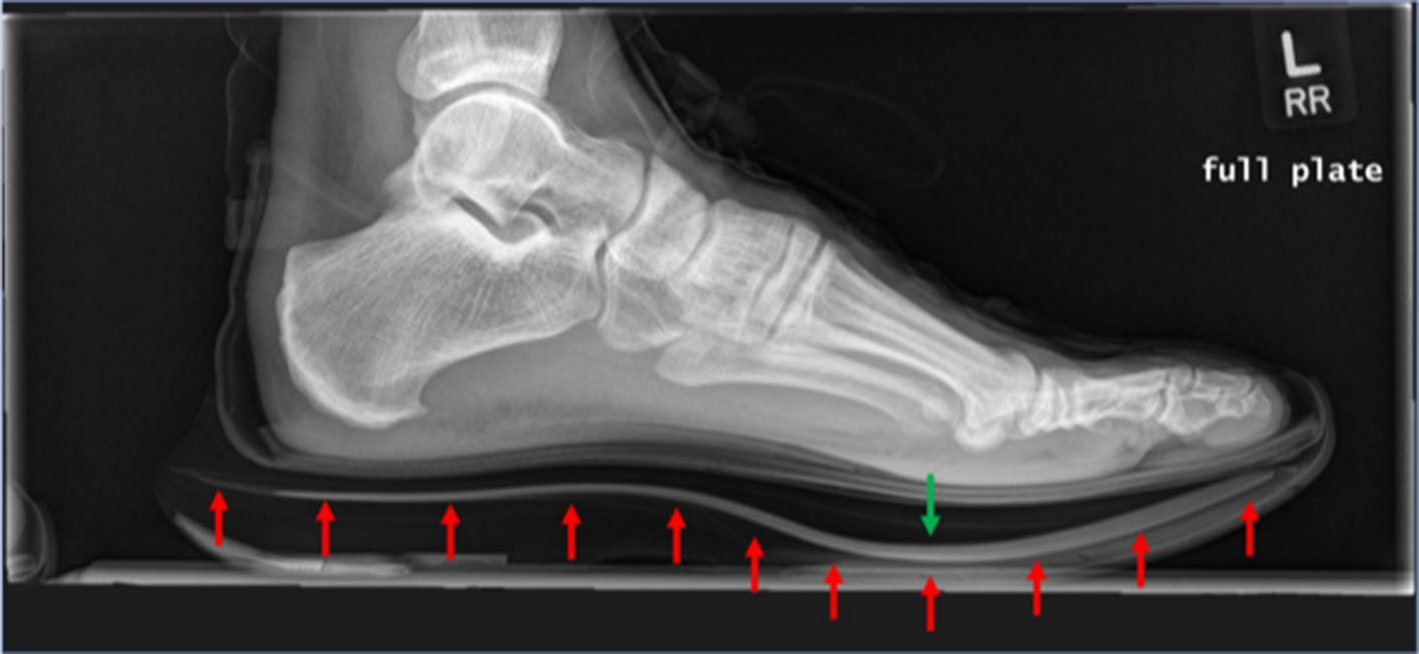
Lateral X-ray of a runner's left foot in a carbon-plated running shoe. Red arrows outline the embedded plate. The green arrow shows the fulcrum point of the plate. Note the relation of the curvature of the plate to the metatarsal locations (metatarsal phalangeal joints)

The sport of running has seen recent changes in training and competition with the use of an embedded carbon fiber plate (CFP) within the midsole of footwear [1]. The CFP spans and is embedded into the midsole inside a compliant and resilient foam [1] (see example in Fig. 1). The early prototypes were worn by elite marathoner Eliud Kipchoge who ran a sub-2-h marathon using CFP footwear in artificial conditions including closed loop circuit and pacemakers [2]. Concerns about fairness in sport were evaluated by World Athletics and resulted in new rules stating that the combination of a single CFP and responsive foam midsoles was permissible for use if not exceeding 25 mm of sole thickness for track (≥ 800 m) and 40 mm for road running (“Athletics Shoe Regulations”, effective from 1 January 2022) [3]. The footwear industry has continued to incorporate this technology into running shoes. Since the introduction of CFP shoes into competition starting in 2016, every world record from 5000 m to marathon distance has been eclipsed by competitors using this new technology [4]. Additionally, sports science has validated the performance benefits of CFP combined with compressive foam midsole compared to earlier footwear used for training and competition [5–8].

## Biomechanics of Carbon Fiber Plate Footwear

The use of CFP footwear during training and competition has been shown to introduce novel biomechanical demands on the foot and lower extremities. The biomechanical differences between a novel CFP footwear compared to standard competitive running footwear have been previously evaluated in competitive male runners [9]. In this investigation, runners using CFP footwear were observed to have decreased cadence and correspondingly longer steps as well as a longer flight time [9]. Furthermore, peak vertical ground reaction forces and the vertical impulse per step were higher in runners using CFP footwear. No changes in knee or hip mechanics but differences in ankle and metatarsophalangeal joint mechanics were observed in runners using CFP footwear [9]. The authors also described that peak ankle dorsiflexion during stance, and peak ankle moments were reduced and lower negative and positive ankle work were observed in CFP over standard competitive footwear during running. These results suggest that more energy was stored in the midsole and less in the muscles and tendons of the ankle [9]. A biomechanical explanation of these findings is that the CFP increases longitudinal bending stiffness of the footwear and, thus, is associated with reduced dorsiflexion of the metatarsophalangeal joints before take-off accompanied by an altered energy storage and return [9]. This suggests CFP footwear may store and return more energy compared to prior standard footwear. Observed improvements in running economy may result from energy return from compression of cushioning material and the lever effects of the ankle mechanics considering the curve of the CFP and a higher toe spring [9, 10]. The CFP has been proposed to create a “teeter-totter effect” that moves forces anteriorly in the foot during the propulsive phase [10]. Importantly, this is not supported by experimental data, which show no difference in the center of pressure progression [9]. The midsole cushioning may also contribute to improvements in running economy, as shown in earlier work [11]. However, the compressive foam would be expected to contribute to a return of energy in the form of vertical displacement and, thus, may be dependent on the footstrike pattern [12].

## Biomechanical Influences Associated with Bone Stress Injuries

The change in foot and ankle mechanics introduced by CFP footwear may contribute to the risk of injury. Bone is an adaptable tissue that responds to changes in demands including those resulting from footwear. For example, a study demonstrated that the gradual introduction of minimalist footwear over 10 weeks resulted in changes on magnetic resonance imaging (MRI) concerning metatarsal bone stress injury (BSI) in a population of runners previously habituated to standard footwear [13]. By extension, the use of CFP footwear could be expected to generate novel stress to bone. BSI represents an overuse injury that is the result of localized failure of bone from cumulative loading and can progress to the development of stress fracture [14]. Navicular BSIs are classified as a high-risk location for injury as some of these injuries may not effectively heal with non-surgical measures [14]. While navicular BSIs are described in older populations of collegiate and professional athletes [15], these injuries have also been observed in youth athletes [16–18]. Prior studies on biomechanical risk factors associated with navicular BSI are retrospective and include reduced ankle dorsiflexion and subtalar range of motion [18], higher peak rearfoot eversion and range of motion [19], both cavus and planus foot types [20], and plantar displacement of navicular and cuneiforms with narrowing of the medial aspect of the talonavicular joint [21]. The navicular bone receives unequal forces from the first and second metatarsocuneiform joints [22] that create shear stress over the central third of the bone, corresponding to a region of reduced blood supply [23], and a common site for navicular BSI. A grading system of navicular BSIs developed by Saxena and Fullem is commonly used to guide evaluation and management based on CT findings and inform surgical decision making [20, 24].

## Case Series of Navicular Bone Stress Injuries in Runners Using Carbon Fiber Plate (CFP) Footwear

This case series reflects clinical observations in five patients presenting with foot pain and diagnosis of navicular BSI who were using CFP footwear at the time of injury. Given the high rate of adoption of CFP footwear in track and field, understanding potential associated health concerns is important for athletes and healthcare providers.

### Case 1

**Fig. 2:**
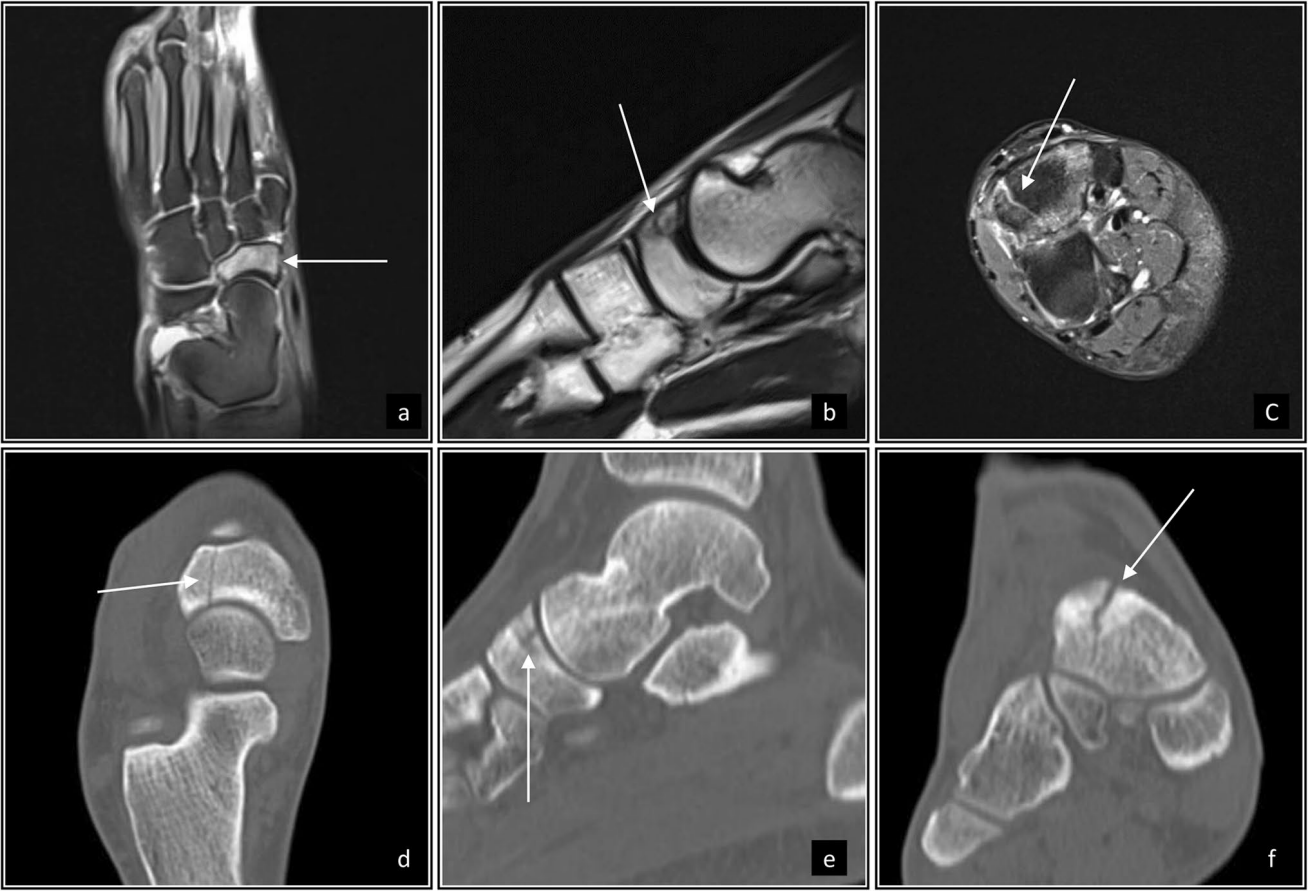
Images from Case 1. Sagittal (**a**), coronal (**b**), and long axis (**c**) on T2 fat-suppressed sequences on magnetic resonance imaging demonstrate vertically oriented stress fracture. Corresponding views on CT visualize the fracture orientation (**d**–**f**) and classify as navicular Type III stress fracture [20]

A 17-year-old male junior elite steeplechase runner was using CFP shoes for interval sessions on the track prior to a race. The athlete felt severe midfoot pain directly after a 3000 m steeplechase race. He had no relevant history of BSIs and had been using different types of carbon-plated shoes for 2 years (completing approximately 1000 km of total running in this footwear). Plain radiographs were performed immediately after the race and the athlete was cleared to continue sports participation. Due to persistent pain over the following 5 weeks, he presented at an outpatient clinic and was diagnosed with a navicular stress fracture. The injury was managed by sports restrictions (no cast immobilization, no weight-bearing restrictions). Six weeks later (at 11 weeks after the inciting race), a follow-up MRI was obtained and did not demonstrate visible bony consolidation (Fig. 2a–c). However, the athlete was pain free and was allowed a gradual return to sports. However, with persistent non-union of the fracture, he was transferred to a specialist with advanced knowledge in foot injuries and sports medicine. A weight-bearing CT revealed a stable Type III navicular stress fracture [20] with the absence of bony consolidation (Fig. 2d–f). After an interdisciplinary case conference with the patient, shared decision-making was applied, and he completed a gradual load buildup on an anti-gravity treadmill at 75% of body weight, and he ultimately returned to land-based running. Despite a follow-up CT scan revealing that the fracture line was still present, the athlete continued to run without pain.

### Case 2

A 17-year-old female junior elite middle-distance runner was using CFP shoes exclusively for interval sessions on the track. She had been using carbon-plated shoes for about 6 months over 100 km. She experienced pain in the midfoot after a track session wearing CFP shoes. The runner had a previous history of a navicular BSI in the same foot 2 years earlier that was treated conservatively. On evaluation, she was noted to have bilateral pes planovalgus. An MRI after the inciting event revealed a Type 0.5 (“stress reaction”) of the navicular bone [20]. She was initially treated with 6 weeks of non-weightbearing in an AirCast. Repeat MRI obtained 6 weeks after the initial diagnosis showed a reduction of edema but still a stress reaction leading to 2 more weeks of non-weightbearing. After a total of 8 weeks of non-weightbearing the athlete started cross-training on an Alter-G treadmill (initially with 70% of body weight) and was back to normal and pain-free training 15 weeks after the initial diagnosis.

### Case 3

**Fig. 3:**
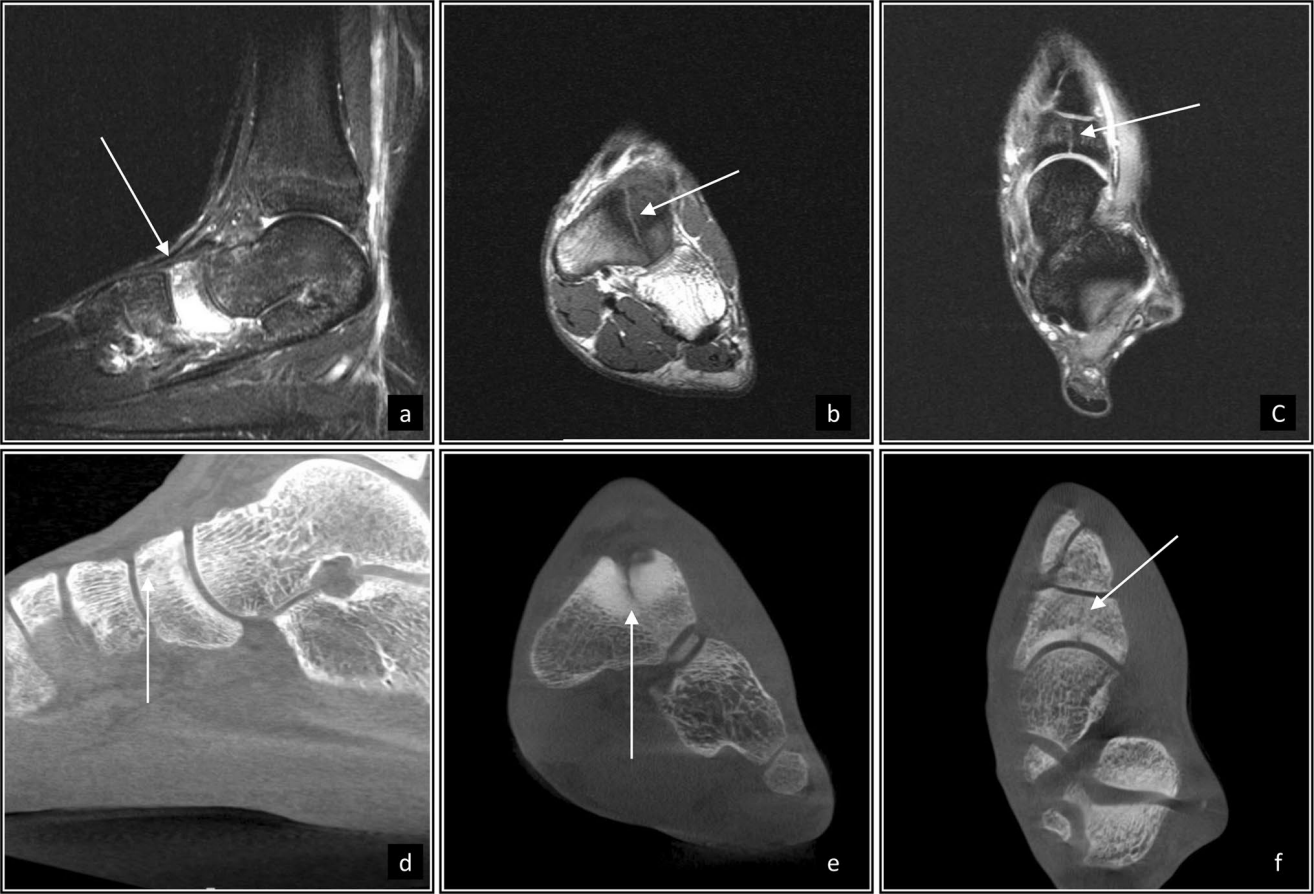
Images from Case 3. Long axis (**a**), sagittal (**b**), and coronal (**c**) on T2 fat-suppressed sequences on magnetic resonance imaging demonstrate vertically oriented stress fracture. Corresponding views on CT visualize the fracture orientation (**d**–**f**) extending through both plantar and dorsal cortices and classify as navicular Type III stress fracture [20]

An 18-year-old female elite 3000 m steeplechase runner was racing a 10 km road race in CFP racing shoes. The race was the first time that she had used the new CFP shoes. The week after the race she experienced foot pain localized at the forefoot with associated minimal swelling and was unable to walk pain-free in the following days. Without medical consultation, she went back to running and experienced trauma from acute supination in the same foot leading to medical consultation. Resulting from this consultation at 4 weeks after the race and 1 week following trauma, an MRI was obtained that revealed a navicular BSI, and a subsequent CT scan confirmed the presence of a Type III navicular stress fracture (Fig. 3). She was subsequently treated with non-weightbearing for 4 weeks in a walker. Afterwards, she initiated strength exercises and cross training on a cycle ergometer. Seven weeks from the time of CT, she attempted to run but experienced pain at level 4/10 on a numeric rating scale. Following one additional week off from running, she was able to return to running pain-free.

### Case 4

A 38-year-old male elite triathlete competed in a half-marathon (13.1 miles/21.1 km) in CFP shoes. The shoe he wore had not been used in any significant training or racing prior. Towards the latter portion of the race, he experienced midfoot pain, and upon completion, was unable to walk pain-free. He had minimal swelling, pain localized to the “N-spot” and experienced throbbing at night. He had a previous history of a navicular BSI in the same foot, treated non-operatively 18 years prior as a collegiate steeplechaser. He also had a history of a navicular BSI in the contra-lateral foot treated operatively 6 years prior. He had a stable foot structure and normally did not wear foot orthoses. Due to his prior history, a CT scan was obtained, which revealed a Type II navicular BSI, and the patient underwent open reduction and internal fixation, and went on to successful healing.

### Case 5

A 36-year-old male elite triathlete ran a 22-mile training run in preparation for a marathon race 4 weeks later. He had only run in the CFP shoes two to three times prior and for much shorter distances. He developed midfoot pain immediately after the inciting run, with similar symptoms to the case above (limping, pain at the “N-spot”, throbbing at night), but no swelling. He had no prior history of a navicular BSI. The athlete normally wore custom foot orthoses but did not use these in his racing shoes. Due to the short time-span of his upcoming race, a CT scan was ordered and was negative for a fracture. He was diagnosed with a Type 0.5 navicular BSI (“stress reaction”) and treated with a below-knee boot, focused extracorporeal shockwave therapy (at 0.40 mJ/mm^2^ for 2000 pulses at the “N-spot”) and electromagnetic transduction therapy (9000 pulses at power level 8, 8 Hz). This treatment was repeated 1 week later, and since he was pain-free, he discontinued the boot. He was allowed cross-training on a stationary bike and swimming after diagnosis. He started training on an anti-gravity treadmill 10 days after initiating treatment at 70% body weight. He was able to run on land approximately 12 days prior to his marathon, and he completed the marathon pain-free.

## Discussion

The purpose of this Current Opinion article is to describe both running performance benefits and potential associations of BSI in runners using CFP footwear. We illustrate this with a series of navicular BSIs in two discrete cohorts including a population of junior elite track and field athletes in Europe and two older athletes competing in endurance events in North America. In all cases, athletes developed acute pain during or after running in CFP footwear. Differences in time to diagnosis and management reflect the relative experience of the healthcare providers who initially evaluated each athlete. A prior study related that the time to reach an accurate diagnosis for navicular stress injuries is almost 9 months [20]. Recognizing possible associations of navicular BSI in runners presenting with vague midfoot or ankle pain who use CFP footwear may be important to identify this high-risk injury. A previous study of 139 elite tennis players reported stress fracture incidence of 12.9%, of which 27% were located in the tarsal navicular [25]. A previous article reports navicular stress fractures to be 35% of all stress fractures [26]. The true incidence may be hard to estimate since many of these injuries go undiagnosed for long periods (on average over 8 months, as data from a large series and a systematic review suggest [24, 26]).

Each case presented involved the use of CFP footwear with a compressible foam midsole designed to improve running economy. The mechanism for injury in each case cannot be determined due to limitations of a case report format and lack of studies to describe the changes in lower extremity biomechanics between forms of training and racing footwear in both sexes [5]. The athletes include a mix of sex, age, use of CFP footwear and primary competition events. Use of custom orthotics and prior history of BSI in athletes could influence injury risk. Two athletes competed in the steeplechase event and prior work has demonstrated higher vertical ground reaction forces with hurdling and water jump landings compared to treadmill running [27].

Based on prior studies describing risk factors for navicular BSI [18, 19, 22], it is plausible that shoes with a compressive foam midsole may allow for increased plantar displacement of the navicular and cuneiform bones and modified forces to the hindfoot. As discussed earlier, multiple biomechanical variables may change using CFP footwear compared with other types of competition shoes. Behaviors of the athlete in their use of these shoes for training and competition may also explain novel demands on the foot, including training at faster velocities, which would be expected to increase skeletal loading [28].

Currently, sports governing bodies permit the use of CFP footwear, and many runners are using these shoes with the aim of enhancing performance. Our case series is the first published cohort to document the potential associated risk of navicular BSI using this new footwear. Athletes choosing to wear CFP footwear should recognize the development of pain, particularly over the navicular bone, anterior ankle or midfoot region, which may represent a more significant injury that requires further evaluation to guide correct treatment. Based on prior evidence of maladaptation following rapid adoption of minimalist footwear use with metatarsal BSI [13], one potential behavioral strategy for runners may be to incorporate CFP footwear gradually into training and competition.

While this is the first report to describe bone stress injuries in association with novel CFP footwear, there are clearly limitations to this work. The development of BSI is often multifactorial [14] and retrospective chart review limits understanding mechanisms for injury. The cases are from two separate cohorts of junior and senior elite from different geographic locations, and it is unclear whether similar injuries have been observed in other populations. The diagnostic testing is described using the Saxena and Fullem classification to provide consistency in descriptors of injury [21].

## Conclusion

This Current Opinion discusses a possible association of BSIs with CFP footwear while recognizing the performance benefits that have been described. Advances in the evaluation and management of BSIs have been extensively published, and highlight the need to identify multiple risk factors for BSIs including those that are modifiable. We recommend further research to better understand whether the association of BSIs with CFP footwear is unique to the described runners in this case series or applies to other running populations. Prior experience with metatarsal BSI with minimalist footwear led shoe companies to develop a more gradual program for transitioning to minimalist shoes; it is plausible that similar advances could be developed by shoe companies, researchers and clinicians to promote safety in sports when using CFP footwear. Further discussions are expected, and both sports industry and sports federations have a duty to respect the guidance and advice of medical professionals. The excitement surrounding this new technology due to faster running times is palpable for both athletes and the sports medicine community. We hope this article helps to guide better recognition of medical issues related to CFP footwear, appropriate use of this new technology, and safety for our athletes.
